# Distinct Age-Related Epigenetic Signatures in CD4 and CD8 T Cells

**DOI:** 10.3389/fimmu.2020.585168

**Published:** 2020-11-11

**Authors:** Bin Hu, Rohit R. Jadhav, Claire E. Gustafson, Sabine Le Saux, Zhongde Ye, Xuanying Li, Lu Tian, Cornelia M. Weyand, Jörg J. Goronzy

**Affiliations:** ^1^ Division of Immunology and Rheumatology, Department of Medicine, Stanford University, Stanford, CA, United States; ^2^ Department of Medicine, Palo Alto Veterans Administration Healthcare System, Palo Alto, CA, United States; ^3^ Department of Biomedical Data Science, Stanford University, Stanford, CA, United States

**Keywords:** ribosomal proteins, chromatin accessibility, epigenetics, T-cell, aging, T-cell homeostasis

## Abstract

Healthy immune aging is in part determined by how well the sizes of naïve T cell compartments are being maintained with advancing age. Throughout adult life, replenishment largely derives from homeostatic proliferation of existing naïve and memory T cell populations. However, while the subpopulation composition of CD4 T cells is relatively stable, the CD8 T cell compartment undergoes more drastic changes with loss of naïve CD8 T cells and accumulation of effector T cells, suggesting that CD4 T cells are more resilient to resist age-associated changes. To determine the epigenetic basis for these differences in behaviors, we compared chromatin accessibility maps of CD4 and CD8 T cell subsets from young and old individuals and related the results to the expressed transcriptome. The dominant age-associated signatures resembled hallmarks of differentiation, which were more pronounced for CD8 naïve and memory than the corresponding CD4 T cell subsets, indicating that CD8 T cells are less able to keep cellular quiescence upon homeostatic proliferation. In parallel, CD8 T cells from old adults, irrespective of their differentiation state, displayed greater reduced accessibility to genes of basic cell biological function, including genes encoding ribosomal proteins. One possible mechanism is the reduced expression of the transcription factors YY1 and NRF1. Our data suggest that chromatin accessibility signatures can be identified that distinguish CD4 and CD8 T cells from old adults and that may confer the higher resilience of CD4 T cells to aging.

## Introduction

With extended lifespans, the number of older individuals steadily increase worldwide. Age is the major risk factor for many diseases, and thus age-associated diseases are becoming a growing public health concern. Aging of the immune system induces a decline in immune competence, resulting in increased vulnerability to infectious diseases and diminished responses to vaccination, while in parallel causing a state of generalized inflammation (1–3). The high morbidity and mortality from influenza infection in the older population, despite the implementation of vaccination programs, has continued to be a prime example illustrating the defective adaptive immune system (4). Even more drastically, the COVID-19 pandemic, with its high fatality rate in the older population, has documented the overall ineffectiveness of the aged immune system. Since SARS-CoV-2 is a newly emerged virus, there is no pre-existing immunity and the adaptive immune response has to rely on the naïve lymphocytes. Thus, the COVID-19 infection has underscored how defective primary immune responses are with advancing age.

Aging causes a functional decline at the cell, organ and organism level involving numerous pathways, commonly summarized as the hallmarks of aging (5). While there is not a single hallmark that is more important than others, the ability to replenish a cellular system is a prerequisite of healthy aging. A failure in replenishment is generally attributed to stem cell aging, but has special meaning for T cells with the involution of the thymus already beginning in early life (6). T cell replenishment in humans rely on homeostatic proliferation and survival of existing T cells. Even in young adults, less than 20% of T cell generation derives from thymic production, which further dwindles to less than 1% in middle-aged individuals (1, 7, 8). To keep immune competence with healthy aging, homeostatic proliferation needs to be effective in maintaining compartment sizes of naïve and memory cell populations, unbiased to not compress T cell receptor (TCR) diversity, while avoiding cellular differentiation (1, 9). These challenges are met in healthy older individuals to a variable degree. In healthy individuals, the number of available TCR in the naïve compartment declines at least two-fold for both CD4 and CD8 T cells, but stays sufficiently diverse to respond to the plethora of antigenic peptides (2, 10). Quantitatively, the naïve and, to a lesser degree, memory CD4 T cell compartments modestly shrink. In contrast, naïve CD8 T cells experience a large loss with age, while memory CD8 T cells preferentially develop into end-differentiated effector T cells (11, 12). In fact, the low frequency of CD8 naïve T cells in the peripheral blood is the most consistent immune aging marker (13–15). Compartment shrinkage is associated with increased heterogeneity in clonal sizes for naïve CD8 compared to naïve CD4 T cells, at the extreme presenting as oligoclonal T cell expansions (10). The cause of the better resilience of CD4 T cells to the aging process is unclear, however, epigenetically, the major age-associated changes in peripheral blood mononuclear cells (PBMC) were attributed to CD8 T cells (16). Possible, not mutually exclusive, mechanisms include poorer survival of CD8 T cells and a lesser ability to maintain quiescence. Indeed, in the mouse, naïve CD8 T cells tend to differentiate into virtual memory T cells more readily than CD4 T cells indicating that they are more easily driven into differentiation (17, 18). Whether virtual memory cells exist in humans is not clear, but our group and others have demonstrated that naïve and central memory CD8 T cells from old adults exhibit epigenetic signatures indicative of a more differentiated state than those from young individuals (19). Moreover, these studies revealed an age-related reduction in accessibility to promoters of genes that are involved in basic cellular maintenance, which may indicate a poorer survival ability. It is currently unknown whether CD4 T cells also display these age-associated signatures; such data could provide insights into mechanisms conferring CD4 T cell resilience to aging compared to CD8 T cells.

Here, we determined chromatin accessibility in CD4 T cell subsets from young and old individuals using ATAC-seq, then integrated and compared these data with previously generated epigenetic profiles of CD8 T cell subsets (19) and related these epigenetic data to differences in the transcriptomes of naïve CD4 and CD8 T cells. Age-associated changes were quantitatively and qualitatively different for the two subsets. The chromatin landscapes of both CD4 and CD8 T cell subsets from old adults exhibited greater accessibility to bZIP family transcription factors compared to T cells from young adults, indicating the development of a more differentiated state with age. Moreover, CD4 naïve and CM T cell subsets appeared overall less differentiated than their CD8 T cell counterparts. More importantly, with age, CD4 T cells largely maintained accessibility to gene-regulatory regions encompassing NRF1 and YY1 transcription factor (TF) motifs that close in CD8 T cells and that are associated with defects in basic cellular maintenance, including reduced ribosomal protein expression. Thus, CD4 T cell resilience is likely mediated by resistance to age-induced differentiation and preservation of basic cellular maintenance functions.

## Materials and Methods

### Study Population and Cells

Peripheral blood and leukapheresis samples from 22 blood donors younger than 35 years and 24 donors older than 65 years, de-identified except for age range, were purchased from the Stanford Blood Center. In addition, peripheral blood samples were obtained from 15 volunteers, who did not have evidence for an acute or uncontrolled chronic disease and who did not have a history of an autoimmune or a malignant disease. T cells were isolated directly from blood with Human T Cell Enrichment Cocktail (STEMCELL Technologies, Canada). CD4 naïve (CD3+CD4+CD62L+CD45RA+CD28+), central memory (CD3+CD4+CD62L+CD45RA–CD28+), and effector memory (CD3+CD4+CD62L-CD45RA–CD28+) T cells were further purified by fluorescence activated cell sorting (FACS) using a BD Aria 3 cell sorter.

### qPCR

Naïve CD4+ and CD8+ CD62L+CD45RA+CD28+ T cells were isolated by FACS followed by RNA extraction using RNeasy Plus Micro Kit (Qiagen). cDNA was synthesized with reverse transcriptase (Promega). Transcripts of target genes were quantified by quantitative PCR on the ABI 7900HT system (Applied Biosystems). Primers used for qPCR were as follows: *NRF1*, F-AGGAACACGGAGTGACCCAA, R- TATGCTCGGTGTAAGTAGCCA; *THPOK*, F-GTCTGCCACAAGATCATCCA, R- TCGTAGCTGTGCAGGAAGC; *β-ACTIN*, F- ATGGCCACGGCTGCTTCCAGC, R- CATGGT GGTGCCGCCAGACAG. *Ct* values were normalized to *β-ACTIN*. Results are shown as relative expression compared to the average expression in CD4 T cells from young individuals.

### Flow Cytometry

The expression of YY1 in naïve (CD45RA+CD62L+) CD4 and CD8 T cells were detected by flow using Cytofix Buffer and Perm Buffer III (BD Biosciences) according to the manufacturer’s protocol. Briefly, T cells were stained with anti-CD4, anti-CD8, anti-CD45RA, anti-CD62L antibodies and LIVE/DEAD™ Fixable Violet Dead Cell Stain Kit followed by standard fixation and permeabilization (BD Biosciences) and stained with antibodies to YY1. Data were collected with a BD LSR Fortessa and analyzed by FlowJo software (version 10.5.3).

### ATAC-Seq and Downstream Analysis

50,000 CD4 naïve, central memory (CM) and effector memory (EM) T cells were isolated by FACS, followed by standard ATAC-seq protocol described previously (20). Reads were trimmed with in-house scripts and aligned to hg19 using Bowtie2. Reads aligned to sex chromatin and reads with mapping quality of less than 20 were excluded. Pre-processed reads were assigned to peaks using the Rsubread package (21). Peaks shared by at least two samples were selected and adjacent peaks were merged if overlapped by at least 50%. The read counts were normalized by combining voom observational-level weights with sample-specific quality weights along with accounting for random effects defined by donors and subsequently applying a robust regression linear model using limma. Differentially accessible peaks were then determined by setting up model contrasts for comparisons of groups (consisting of age young/old, lineage CD4/CD8 and subsets naïve/CM/EM) (22). Contrasts accounting for differences in aging across all differentiated subsets were determined by combining subset specific differences e.g., (naïve/old - naïve/young) + (CM/old - CM/young) + (EM/old - EM/young).

Transcription factor binding site enrichment was calculated with HOMER, comparing peaks more open in one cohort (corrected p-value < 0.05) with peaks that were not found to be more open in that comparison. Multiple hypothesis correction was performed with the Benjamini-Hochberg procedure. For each transcription factor family, only the highest ranked member based on the significance levels of enrichment is shown.

Genes closest to the differentially accessible sites were identified by HOMER annotatePeaks.pl command with default parameters. Biologic process enrichment analysis of genes closest to the differentially accessible sites was performed using the DAVID Functional Enrichment Tool; EASE Score (a modified Fisher Exact p-value) was used to assess the enrichment with scores less than 0.1 considered as enriched. Peaks were visualized by IGV, the y-axis shows scale of insertions per million reads in peaks. Read counts were further smoothed using a 100-bp-radius boxcar kernel for better visualization. Gene and TSS annotations were based on the RefSeq and GENCODE databases.

### RNA-Seq and Downstream Analysis

Naïve CD4+ and CD8+ CCR7+CD45RA+CD28+ T cells and CCR7+CD45RA- CM and CCR7-CD45RA- EM CD8 T cells were isolated from 5 young and 6 old individuals by FACS, followed by RNA extraction with RNeasy Plus Micro Kit (Qiagen), RNA quality was determined with a 2100 Bioanalyzer (Agilent Technologies). Libraries were prepared using Ovation Human FFPE RNA-Seq Library Systems (NuGEN, San Carlos, CA), quantified with a KAPA Library Quantification Kit (Kapa Biosystems, Wilmington, MA) and sequenced on an Illumina NextSeq 500 at the Stanford Functional Genomics Facility.

RNA-seq reads generated from the sequencing runs were analyzed using the nf-core pipeline to determine read counts mapped to genes in GRCh37 genome (23). The data were further analyzed using Bioconductor packages edgeR and CQN after removing genes with low counts. To reflect the experimental design, a mixed model approach was applied, modeled upon sample “Group” as defined above for ATAC-seq and setup as (~ 0 + Group). The downstream analysis to identify differentially expressed genes was performed as described in Chen et al. (24), followed by combining voom observational-level weights with sample-specific quality weights along with accounting for random effects defined by donors and subsequently applying a least squares linear model using limma. Differentially expressed genes were determined by setting up pairwise comparisons between model contrasts.

### Principal Component Analysis

Principal Component Analysis (PCA) was run on the top 5000 most variable peaks in ATAC-seq and the top 500 most variable genes in RNA-seq. The DESeq2 package in R was used to plot the PCA with specifying the number of top genes/sites to be considered. The rowVars function in R was used to calculate variance estimates for each row in a matrix. For ATAC-seq analysis, the top 500 peaks loading a principal component (PC) were used for transcription factor binding site predictions using HOMER.

### Pathway Analysis

Differentially accessible ATAC-seq peaks were associated to genes using GREAT (25). For peaks assigned to multiple genes, only the gene closest to the peak was chosen. The gene list obtained by this method was used in Ingenuity Pathway Analysis to determine significantly enriched canonical pathways. Likewise, enrichment for canonical pathways was determined for differentially expressed genes derived from RNA-seq data.

### Statistical Analysis

Statistical analysis was performed using GraphPad Prism8.4. (GraphPad Prism) using two-sided t-test, one-way ANOVA, or Mann-Whitney test, for group comparisons as appropriate. To compare PC levels between CD4 and CD8 T cells, we used pair-t test stratified by cell lineage and age (naïve/young, naïve/old, CM/young, CM/old, EM/young, and EM/old). To compare PC levels between old and young participants, we used two-sample t-test stratified by cell type and lineage (naïve/CD4, naïve/CD8, CM/CD4, CM/CD8, EM/CD4, and EM/CD8). In both cases, nonparametric permutation (permuting CD4 and CD8 labeling within a participant and young vs. old participants, respectively) was used to generate the null distribution of test statistics. A p-value of less than 0.05 was considered significant.

## Results

### Age-Associated Epigenetic Changes in CD4 and CD8 T Cells

**Figure 1:**
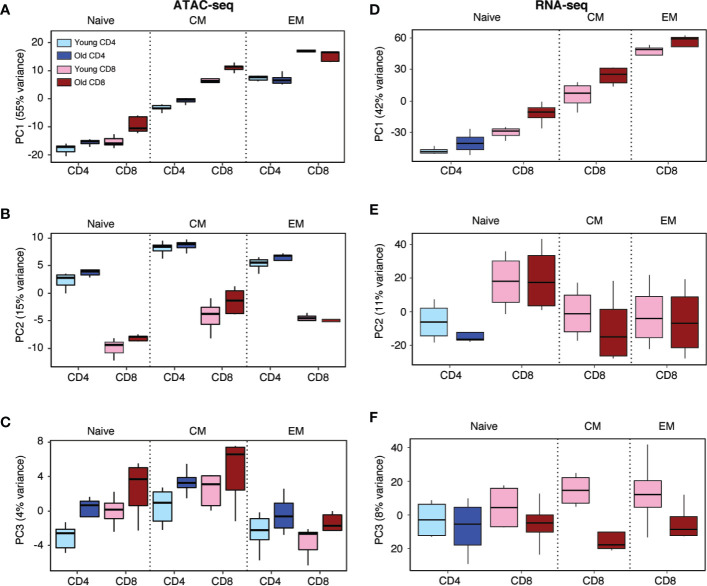
Principal component analysis of CD4 and CD8 T cell subsets from young and old healthy individuals. **(A–C)** Principal component analysis (PCA) of ATAC-seq data of CD4 and CD8 naïve, central memory (CM) and effector memory (EM) T cells. Boxplots of PC1 **(A)**, PC2 **(B)**, and PC3 **(C)** of 5000 most variable sites of accessibility are shown for indicated T cell subsets from young and old individuals. PC1 segregated by differentiation (p = 0.006) and to a lesser degree by age (p = 0.042), PC2 by T cell lineage (p = 0.016) and PC3 trending to by age (p = 0.12). **(D–F)** PCA of RNA-seq data of naïve CD4 and CD8 naïve and CD8 CM and EM T cells from young and old individuals. Boxplots of PC1 **(D)**, PC2 **(E)**, and PC3 **(F)** of 500 most variable genes are shown for indicated T cell subsets from young and old individuals.

To determine whether the age-associated differences in population dynamics of CD4 and CD8 T cell subsets are reflected in their epigenetic states, we generated ATAC-seq libraries of purified CD4 naïve, central memory (CM) and effector memory (EM) T cells from 6 healthy donors under age 35 and 4 healthy donors over 60 years old. Frequencies of T cell subsets in these ten individuals were consistent with previous findings by us and others that loss of naïve CD8 T cells with age is more pronounced than that of naïve CD4 T cells (**Supplemental Figure 1**) (14, 15). These chromatin accessibility profiles of CD4 T cell subsets were then compared with those of CD8 T cells previously published from our group (19). Peak sizes reflecting accessibility from all samples were pooled and principal component analysis (PCA) of the 5000 most variably accessible sites was performed. The scatter plots in **Supplemental Figure 2** shows PC1 vs. PC2 of all samples with differentiation states indicated by different colors and lineage by different symbols. PC1 (55% of variance) ordered T cell subsets according to differentiation state (EM > CM > naive), PC2 (15% of variance) separated by lineage. Results of all 12 subgroups (three differentiation states of CD4 and CD8 T cells, each from young and old individuals) are summarized as box plots (**Figure 1**). As shown with PC1, all four subgroups (CD4 and CD8 T cells, each from young and old individuals, showed the same pattern of epigenetic changes with differentiation, as determined by stratified test, i.e., comparisons were stratified by cell type and age groups, a trend test was performed within each group and results were then combined (p = 0.006, **Figure 1A**). Moreover, PC1 for CD4 naïve and CM cells from older individuals was higher than those from young individuals. PC1 for CD8 T cell subsets showed the same relationship. The probability that this order for CD4 (or CD8) T cells, i.e., CM/old > CM/young > naive/old > naive/young, occurred by chance is only 0.042, suggesting that age-related epigenetic changes are similar to those of differentiation, even without changes in classical cell surface phenotypes. In addition, PC1 for CD8 T cells is significantly larger than that for CD4 cells based on paired comparisons stratified by differentiation state and age (EM/old, EM/young; CM/old, CM/young; naïve/old, naïve/young) (p = 0.035).

PC2 mainly segregated samples by T cell lineage across all three differentiation stages and irrespective of age (**Figure 1B**). Specifically, PC2 for CD4 was statistically significantly greater than that for CD8 cells based on paired comparisons as described above (p = 0.016). Differentiation-dependent differences were much smaller and did not have the clear trajectory from naïve over CM to EM T cells. PC3 (4% of variance) segregated by age across both lineages and all three differentiation states. The difference did not reach significance based on two-sample comparisons stratified by differentiation state and lineage (EM/CD4, EM/CD8; CM/CD4, CM/CD8; naïve/CD4, naïve/CD8) (p = 0.12, **Figure 1C**), possibly due to limited sample size. An effect of lineage was only seen for naïve T cells, with PC3 in CD8 T cells higher. Similar to PC2, PC3 in CM was slightly higher in CM than naïve and EM T cells. Taken together, the major segregations were T cell differentiation in PC1, T cell lineage in PC2 and age in PC3. All the aforementioned p-values are two-sided and based on nonparametric permutation tests.

The top 500 peaks loaded for each PC were used for transcription factor (TF) binding site prediction using HOMER motif analysis (**Supplemental Figure 3**). TF motifs enriched in PC1 loading were foremost of the bZIP family, followed by other TFs characteristic of memory and effector T cells including RUNT and T-box families. **Supplemental Figure 3A** shows the top-ranked member of each family (e.g., BATF for bZIP family) with the corresponding significance level of motif enrichment. ETS1 TF motifs dominated PC2 loading (**Supplemental Figure 3B**). Motifs of known lineage-specific TFs including GATA3 and RUNX1 for CD4 T cells were also highly enriched, while ZBTB motifs, recognized by the CD4 cell-specific TF THPOK, were not present in the ten top-ranked family motifs, although still significantly enriched. TF motifs enriched in PC3 loading encompassed a number of TFs, without a single one being dominant or directly related to T cell differentiation (**Supplemental Figure 3C**). Taken together, chromatin accessibility maps showed a high correlation with cell identity in respect to lineage and differentiation state. Age-associated differences can be identified for CD4 and CD8 T cells, which in part indicate a higher degree of differentiation.

PC analysis of the corresponding RNA-seq paralleled the observation described for ATAC-seq (**Figures 1D–F**). RNA-seq data was obtained from naïve CD4 and CD8 T cells from young and old individuals. In addition, we analyzed the transcriptomes of CD8 CM and EM cells from these individuals. The highest variance was again associated with the differentiation state (**Figure 1D**). As also seen in PC1, older age was associated with a shift in the same direction as differentiation. PC3 segregated by age across all three different CD8 T cell differentiation states, but did not show a shift for naïve CD4 T cells. Specifically, PC1 for CD8 is significantly greater than that for CD4 based on paired comparisons stratified by differentiation state and age (naïve/old; naïve/young) (p = 0.035). PC2 of RNA-seq for CD8 was also significantly greater than that for CD4 based on stratified paired comparisons (p = 0.016). However, a clear relationship between transcriptome and T cell lineage was less obvious than for the ATAC-seq data, because we did not extend the study to CD4 memory T cell subsets. PC3 appeared to segregate by age, but again, the difference did not reach statistical significance based on two-sample comparisons stratified by differentiation state and lineage (EM/CD8; CM/CD8; naïve/CD8; naïve/CD4) (p = 0.14). Again, all the aforementioned p-values are two-sided and based on permutation tests. Taken together, the PC analysis of chromatin accessibility and transcriptome suggest that the largest age-related variance is related to a higher differentiation state of CD4 and CD8 T cells from older individuals.

### Differentiation-Related Signatures in Age-Associated Epigenetic Changes

**Figure 2:**
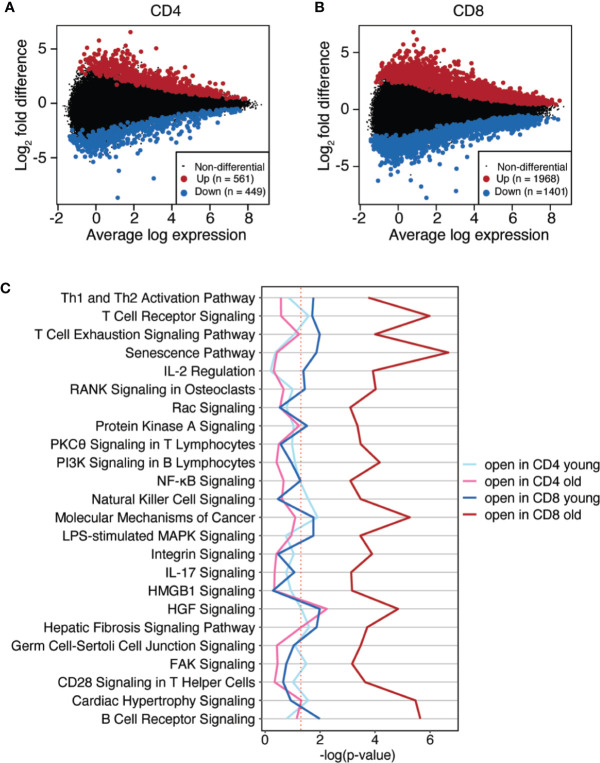
Age-associated differences in chromatin accessibility in CD4 and CD8 T subsets. **(A, B)** Scatter plots (MA plots) of log2 fold differences between young and old individuals versus the mean of normalized counts. Age-related differences across all differentiation subsets were determined by combining naïve, CM and EM subset-specific differences for CD4 **(A)** and CD8 T cells **(B)**. Colored dots [more accessible in old (red) or young (blue)] indicate differentially accessible sites with Benjamini-Hochberg adjusted p-values of <0.05. **(C)** Age-associated, differentially accessible sites were assigned to genes by GREAT. Gene lists were used to identify most enriched canonical pathways by Ingenuity Pathway Analysis. X-axis shows the adjusted p-value, red dash line indicates adjusted p-value of 0.05.

In order to compare differential peak accessibility across ATAC-seq datasets, read counts from ATAC-seq were normalized by applying a robust regression linear model using limma to account for sample-specific quality weights along with random donor effects. Differentially accessible peaks were then determined by setting up a model contrasting group comparisons (22). Age-related differences across all differentiation subsets were determined by combining subset-specific differences, i.e. (naïve/old - naïve/young) + (CM/old - CM/young) + (EM/old - EM/young)” separately for CD4 and CD8 T cells (**Figures 2A, B**).

Using p < 0.05 as cut-off, we found a threefold higher number of sites changing accessibility with age in CD8 T cells (3369 peaks) compared to CD4 T cells (1010 peaks). We used GREAT to associate differentially accessible regulatory regions with their predicted genes. Ingenuity Pathway Analysis was then used to identify canonical pathways enriched in these gene sets (**Figure 2C**). Genes with more accessible regulatory sites in CD8 T cells from older individuals were significantly associated with numerous pathways (adjusted p-values of around 10^-4^), with a common theme of cellular activation. The senescence and the T or B cell receptor signaling pathways reached significance levels of nearly 10^-6^. In contrast to CD8 T cells from older adults, genes with increased accessibility in regulatory regions in CD4 T cells were not enriched for any of these pathways. Only the HGF pathway was significant with an adjusted p-value of less than 10^-2^. Gene sets corresponding to predicted regulatory region more accessible in the young did not show strong enrichments for CD4 or CD8 T cells.

**Figure 3:**
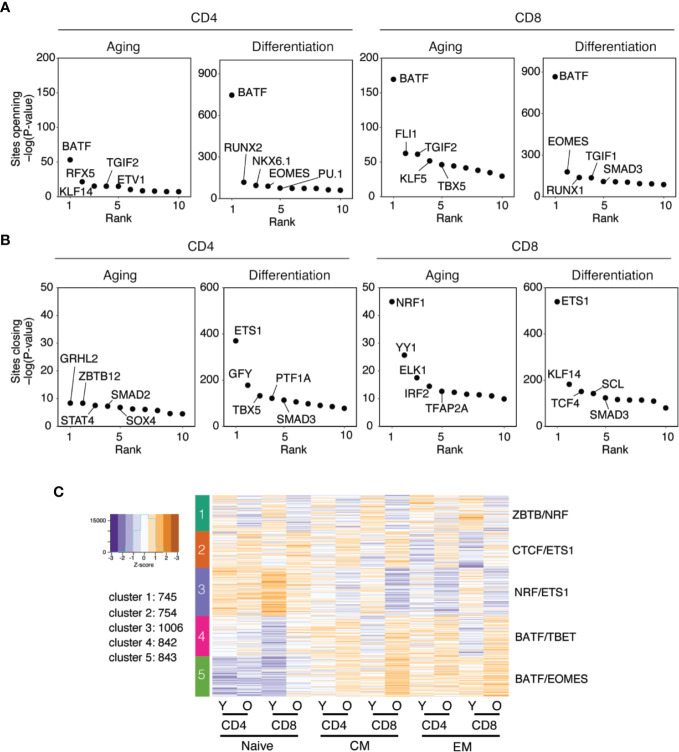
Transcription factor networks in T cell aging and differentiation. **(A, B)** TF motif enrichments at sites more (3A) or less accessible (3B) with age and differentiation in CD4 (left) and CD8 (right) T cells are compared. X-axis shows the rank of the TFs, y-axis the significance level of TF motif enrichment **(C)** *k*-means analysis of sites differentially accessible with age as defined in **Figures 2A, B**. The number of clusters was determined by gap statistics, and results are shown as heat plots with each horizontal line representing a differentially open site (left). Columns represent CD4 and CD8 T cell subsets and colors *z*-scores of openness. Top transcription factor motifs significantly enriched at the sites within each cluster are indicated at the right margin.

To identify TF networks associated with the epigenetic changes in the CD4 and CD8 T cell lineages, we calculated the enrichment of TF binding motifs at sites that significantly differed in accessibility with age, using HOMER motif analysis software. Given the motif similarity for TFs within families, we only show the highest ranked TF within each family (**Figures 3A, B**). The highest enriched motif at sites opening with age was BATF of the bZIP family, in particular for CD8 but also for CD4 T cells (**Figure 3A**). As shown in **Supplemental Figure 3**, T cell differentiation is associated with increasing accessibility to BATF. We identified sites opening with differentiation by comparing EM to naïve T cells and contrasted the motif enrichment at these sites with those gaining accessibility with aging. The main similarity was the enrichment for BATF motifs. Other TFs, associated with T cell differentiation such as T-BET or EOMES (T-box family) and RUNX1 or RUNX 2 (RUNT family), were also affected by aging, but were not included in the top hits and of lesser significance. The correlation between differentiation and aging was less striking for TF motifs at sites losing accessibility. For both CD4 and CD8 T cells, sites closing with differentiation were enriched for ETS1 and, with lesser significance and not top-ranked for CD4 T cells, TCF motifs (**Figure 3B**). Loss in accessibility to ETS1 family members (here represented through ELK1) was also observed for CD8 T cells of older individuals; however, other TF family motifs were more enriched at the closing sites, such as NRF1 and YY1 for CD8 T cells. This pattern in CD8 T cells was distinct from CD4 T cells. These data suggest that the increased differentiation states of naïve and CM T cell subsets with age, as observed in PC1, are mainly driven by the gain in accessibility to bZIP sites exemplified by BATF for both CD4 and CD8 T cells.

To further characterize the age-associated epigenetic changes, we performed *k*-means clustering of the sites that changed with age as shown in **Figures 2A, B**. Results are shown as heat plot for naïve, CM and EM CD4 and CD8 T cells subsets (**Figure 3C**). In addition, for better illustration of cluster-specific features, *z*-scores of each cluster for each sample was summarized as box plot (**Supplemental Figure 4**). Five clusters were formed by gap statistics. To identify which regulatory elements contributed to age-associated differences, we examined each *k*-means cluster for enrichment of TF binding motifs *via* HOMER. Clusters 1 and 2 included sites that were more (cluster 1) or less accessible (cluster 2) in T cells from young adults, independent of the differentiation state. Sites in the remaining three clusters, all correlated with differentiation. Since only sites that significantly differed in accessibility with age were included in the heat plot, the additional shift in accessibility with differentiation again supported the notion that both processes are related, at least for the regulatory regions included in these clusters. Sites in cluster 3 closed with differentiation, more so in CD8 than CD4 T cells. Clusters 1 and 3 were highly enriched for NRF1 motifs. ETS1 motifs, known to close with T cell differentiation, were the top TF motif enriched in Cluster 2 as well Cluster 3. Sites in clusters 4 and 5 opened with differentiation, and accordingly bZIP (BATF) and T-box (T-BET or EOMES) motifs were most significantly enriched at those sites. For all clusters, patterns of age-associated changes were similar for CD4 and CD8 T cells, however, changes of sites in Clusters 3 and 4 were more pronounced for CD8 T cells. Stratification by clusters did not lead to a higher enrichment for functional pathways compared to separately analyzing CD4 and CD8 T cells (**Supplemental Figure 5**). Clusters 1 and 4 did not show convincing enrichments. A relative enrichment for PKA signaling was seen for cluster 2 that included sites with increased age-related accessibility across all differentiation states. Clusters 3 and 5 genes were enriched for numerous signaling pathways. Significance levels were generally not high, and there was not a single pathway or a common denominator of associated pathways that was dominant.

### Age-Associated Changes in the Transcriptome of Naïve CD4 and CD8 T Cells

**Figure 4:**
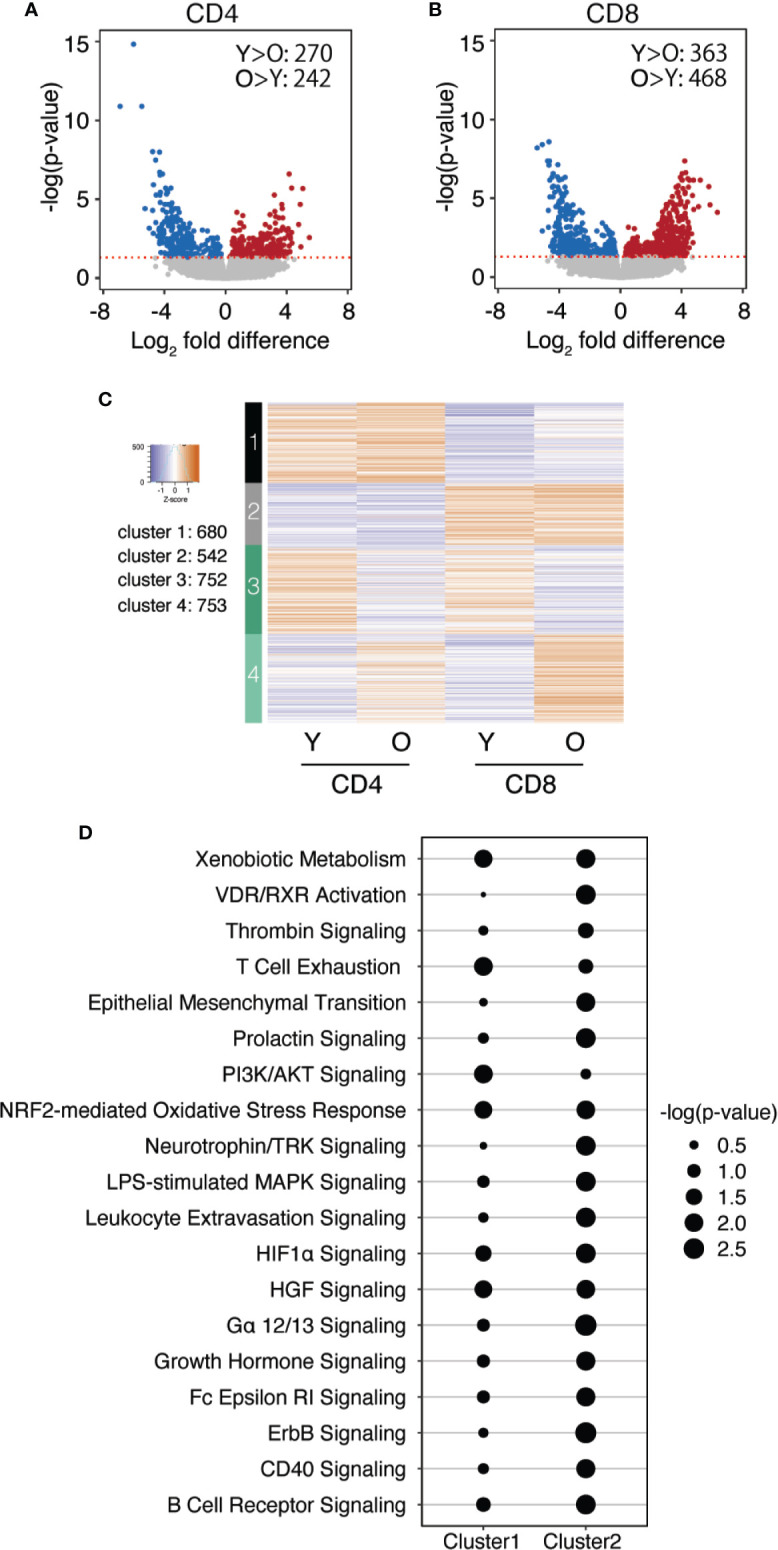
Age-associated transcriptional differences in naïve CD4 and CD8 T cells. **(A**, **B)** Volcano plots show the log2 fold differences between young and old individuals at transcript level comparing naïve **(A)** CD4 and **(B)** CD8 T cells. Colored dots [more expressed in old (red) or young (blue)] indicate differentially accessible sites with Benjamini-Hochberg adjusted p-values of <0.05, red dash line indicates adjusted p value of 0.05. **(C)** Heat plot shows transcript levels as derived from RNA-seq data for all genes that had been linked to differentially accessible sites by GREAT as described in **Figure 2**. Results of *k*-means analysis are shown as heat plots with four clusters, with columns representing CD4 and CD8 naïve T cells from young and old adults and colors *z*-scores of transcripts level. **(D)** Gene sets within each cluster were examined for pathway enrichment using the Ingenuity Pathway Analysis software. Pathways are given for Clusters 1 and 2, with dot sizes indicating the level of significance. Significantly enriched pathways were not identified for Clusters 3 and 4.

To relate the age-associated changes in chromatin accessibility to changes in the transcriptome, we compared naïve CD4 and CD8 T cells from young and old individuals for their transcriptomes. To adjust for the experimental design, we used the mixed model approach as described in the Methods section. Differentially expressed genes were determined by setting up pairwise comparisons between model contrasts. As shown in the volcano plots in **Figures 4A, B**, about an equal number of transcripts were down- or upregulated with age in naïve T cells. Transcriptional changes were more frequent for CD8 than CD4 T cells (831 vs. 512). As shown in **Supplemental Figure 6**, the transcriptional changes in CD4 and CD8 T cells were largely non-overlapping. Clusters 1 and 2 included genes that transcriptionally changed in CD8 T cells with no or only minimal age-related difference for CD4 T cells. Conversely, differences in gene expression as shown in clusters 3 and 4 were largely limited to CD4 T cells. Pathway analysis of the genes in the four clusters did not yield significant enrichments.

To probe the relationship between age-related changes in chromatin accessibility and transcription, we plotted the transcript numbers for naïve T cells of all genes with differentially accessible regulatory regions identified by GREAT as described in **Figure 2C**. As shown in **Figure 4C**, about half of the differentially accessible genes were also differentially transcribed (clusters 3 and 4), while the other half only had an age-associated effect on the accessibility to gene-regulatory regions, but not on the transcriptome itself (clusters 1 and 2). One possible explanation is that these latter genes are differentially poised and will differ in their transcriptomes after activation. Interestingly, the differentially accessible and differentially transcribed genes had the same patterns in CD4 and CD8 T cells (Cluster 3 and 4). In contrast, the genes with only age-associated differential accessibility showed a lineage specific patter, being either transcribed in CD4 (cluster 1) or CD8 T cells (cluster 2). Pathway analysis did not show any enrichment for the differentially transcribed genes from clusters 3 and 4. In contrast, several pathways were identified for genes that were differentially accessible with age, but transcribed in a lineage-specific pattern (**Figure 4D**). Adjusted p-values were in general modest, and pathways were more related to basic cell biological function than T cell biology.

### Comparison of CD4 and CD8 T Cells for Age-Associated Epigenetic Signatures

**Figure 5:**
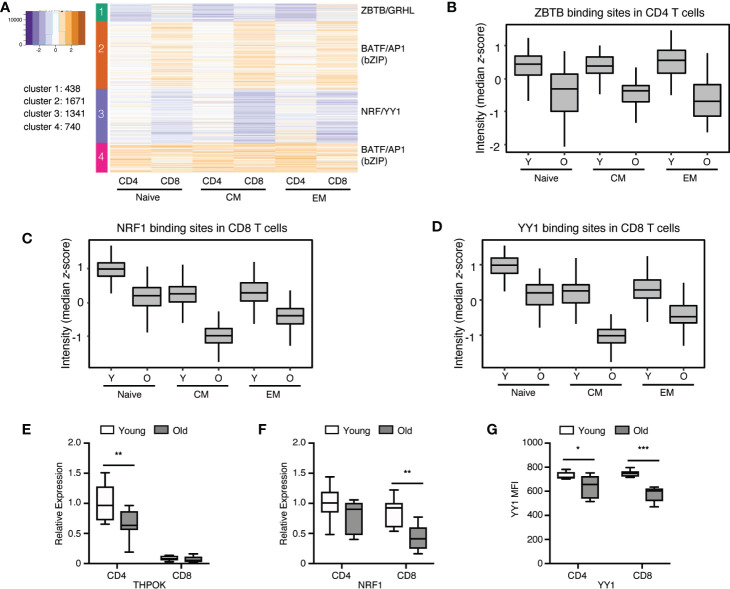
CD4 and CD8 subset-specific changes in chromatin accessibility with age. **(A)** Age-associated differences in chromatin accessibility for CD4 and CD8 T cells were expressed as log2 fold differences and were clustered using *k*-means statistics. Log2 fold differences of accessibility for sites within each cluster are shown as heat plots organized in columns of CD4 and CD8 T cell subsets. Top transcription factor motifs enriched at sites in each cluster, as determined by HOMER, are shown at the right margin. **(B–D)** Box plots show motif intensities for selected TFs, **(B)** ZBTB in CD4 T cells, **(C)** NRF1 and **(D)** YY1 in CD8 T cells, as identified in **(A)**. The motif intensity was calculated based on the *z*-score of the median accessibility for each group. **(E, F)** Gene expression of selected TFs THPOK **(E)** and NRF1 **(F)** in CD4 and CD8 naïve T cells were determined by qPCR. Results are shown normalized to β-actin expression and relative to the mean expression in young CD4 naïve cells. Box plots show data from 8 young and 8 old adults; statistical analysis by two-tailed t-test. **p < 0.01. **(G)** YY1 expression was determined by flow cytometry of naïve (CD45RA+CD62L+) CD4 and CD8 T cells from 8 young and 8 old donors. Comparisons were analyzed by two-tailed t-test, *p < 0.05, **p < 0.01, ***p < 0.001.

In **Figure 3C**, we used *k*-means clustering with gap statistics to cluster peak sizes at all sites that changed with age, irrespectively of whether they were derived from CD4 or CD8 naïve or memory T cells. Cluster formation therefore was driven by variables in addition to age. To focus on age and lineage as the major variables, we calculated the fold difference of peaks comparing young and old and clustered the peaks into four groups based on the fold differences in CD4 and CD8. The results are shown as a heatplot of log2 fold differences for CD4 and CD8 T cell subsets across all differentiation stages in **Figure 5A**, the corresponding *z*-scores of the chromatin accessibilities in young and old adults as box plots in **Supplemental Figure 7**. In general, more sites exhibited larger age-related differences in accessibility in CD8 (1671 and 1341) than CD4 T cells (438 peaks). Group 4 (740 peaks) included peaks with fold differences similar in CD4 and CD8 T cells. Sites gaining in accessibility with age showed an enrichment for bZIP TF motifs including BATF and AP1 irrespective of whether the difference was more dominant in CD4 or CD8 T cells (**Figure 5A** and **Supplemental Figures 8A, B**). In contrast, sites closing with age exhibited different motif patterns for CD4 and CD8 T cells. Group 1 included sites that lost accessibility predominantly in CD4 T cells and were enriched for ZBTB motifs (**Figure 5B**), foremost for ZBTB12, a paralogue of the CD4 lineage-determining TF THPOK (26). In contrast, NRF1 and YY1 binding sites were enriched at sites that closed preferentially in CD8 T cells from older individuals (**Figures 5C, D**).

We examined whether the change in accessibility with age corresponded to reduced expression of the respective TFs. As per RNA-seq data of naïve T cells, transcription of bZIP family members increased with age for both CD4 and CD8 T cells (**Supplemental Figures 9A, B**) (27). Overall, ZBTB family members were not significantly different in CD4 and CD8 T cells from young and old individuals (**Supplemental Figures 9C, D**) (26). However, specific quantification of THPOK transcripts showed a decline in CD4 T cells with age (**Figure 5E**). Expression of NRF1 transcripts (**Figure 5F**) and YY1 protein (**Figure 5G**) was greatly reduced in CD8 from older individuals (p < 0.001), but much less so in CD4 T cells from older individuals (n.s. for NRF1, p = 0.02 for YY1). Taken together, age-related differences in accessibility correlated with differences in TFs expression in CD4 and CD8 T cells that in part were lineage specific.

### Biological Processes Regulated by Sites Selectively Closing in CD8 T Cells With Age

**Figure 6:**
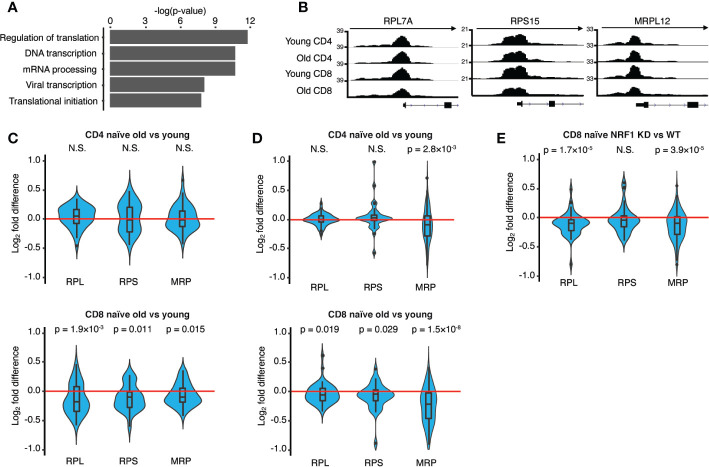
Age-associated effects on ribosome proteins in CD8 T cells at the epigenetic and transcriptional level. **(A)** The top five biological processes enriched in Cluster 3 (**Figure 5A**) as determined by DAVID Functional Annotation Tool. Genes were identified within 10 kb of a differentially accessible region using HOMER. Corresponding analyses for the other clusters are shown in **Supplemental Figure 10**. **(B)** ATAC-seq signal tracks showing chromatin accessibility of representative ribosome protein genes. **(C)** Violin plots showing the fold difference of accessibilities for ribosome protein (large subunit, RPL; small subunit, RPS; and mitochondrial, MRP) comparing naïve CD4 (upper panel) and CD8 T cells (lower panel) from young and old adults, positive values mean more open in the old. Statistical analysis by Wilcoxon rank sum tests. **(D)** Violin plots showing the fold difference of ribosome protein (RPLs, RPSs and MRPs) transcripts comparing naïve CD4 (upper panel) and CD8 T cells (lower panel) from young and old adults, positive values mean more expressed in the old. Transcript data are from RNA-seq of CD4 and CD8 T cells from 5 young and 6 old adults. Statistical analysis by Wilcoxon rank sum tests. **(E)** Violin plots showing the fold differences of ribosome protein (RPLs, RPSs and MRPs) comparing NRF1 knockdown cells to control transfected cells, positive values mean more expressed in NRF1 knockout cells. Transcript data are from naïve CD8 T cells from three young adults transfected with either NRF1 siRNA or control siRNA (dbGaP accession #: phs001187.v1.p1). Statistical analysis by Wilcoxon rank sum tests.

We identified genes corresponding to putative gene-regulatory regions that changed in accessibility in CD4 vs. CD8 T cells by age as defined in **Figure 5A** using the proximity algorithm in HOMER, and subsequently performed DAVID enrichment analysis for biological processes. Genes included in clusters 1 and 4, both more related to CD4 T cell aging, showed minimal enrichment for biological processes (**Supplemental Figure 10**). Genes in cluster 2, more opening in CD8 T cells with age, were significantly enriched for processes pertinent to NF-kB activity. The tracks of few representative genes shown for each cluster illustrate T cell subset-specific differences in accessibility that in part explains the differential age-associated changes. Pathway enrichment was most convincing for genes of cluster 3 that had less accessible region in CD8 cells and that were enriched for processes in mRNA translation and transcription (**Figure 6A**). Accordingly, accessibility to promoters of ribosomal protein genes was reduced in CD8 but not in CD4 cells. Representative tracks are shown in **Figure 6B**. The large ribosomal subunit (RPL) consists of 51 proteins, the small ribosomal subunit (RPS) of 34 proteins and the mitochondrial ribosome (MRP) of 78 proteins (28). Fold difference in peak sizes for all ribosomal protein promoters are shown as violin plots in **Figure 6C**. Overall, peak sizes were significantly lower in naïve CD8 but not CD4 naïve T cells from old individuals. This finding is consistent with the observation that NRF1 and YY1 motifs are enriched at sites in cluster 3 as these TFs regulate the expression of ribosomal protein genes (29).

To examine whether the overall expression of ribosomal genes in CD8 cells declines with age, we analyzed the RNA-seq data of naïve CD4 and CD8 T cells for ribosomal proteins as defined in the HGNC database. As shown in **Figure 6D**, we found ribosomal protein transcripts significantly reduced in CD8 cells from old adults, especially the genes for mitochondrial ribosomal proteins. A similar reduction was seen by analyzing a previously published RNA-seq study of human naïve CD8 T cells (dbGaP accession #: phs001187.v1.p1) shown as **Supplemental Figure 11** (19). In contrast, we only found a lesser, although still significant, reduction of the mitochondrial ribosomal protein transcripts in CD4 cells. To examine whether NRF1 may influence transcript expression of ribosomal proteins, we analyzed RNA-seq data from NRF1-deficient CD8 T cells (dbGaP accession #: phs001187.v1.p1). Knockdown of NRF1 decreased transcripts of ribosomal protein transcripts, which reached significance for mitochondrial and large subunit ribosomal protein transcripts (**Figure 6E**).

## Discussion

In this study, we determined the chromatin landscapes and the transcriptomes of CD4 and CD8 T cells subsets from young and old adults to identify potential mechanisms involved in T cell aging that explain the different resilience of these two lineages to aging. We found that age-associated changes were less pronounced in CD4 than in CD8 T cells. For both CD4 and CD8 T cells, the aging signature had features of differentiation, mostly driven by increased accessibility to bZIP family TFs, such as BATF and, to a lesser extent T-box family TFs, such as T-BET and EOMES. BATF is a pioneer TF, which reshapes the chromatin landscape and facilitates transcriptional programming of effector T cells (30). It also functions as a differentiation checkpoint to orchestrate the gene expression of TCR-dependent transcription factors and gene expression of effector related molecules (31). T-BET is TH1 lineage-determining TF in CD4 T cells, T-BET and EOMES are regulators of CD8 effector and memory cell differentiation (32, 33). Thus, the aging signature is consistent with the interpretation that, although turnover rates are low, T cells cannot completely maintain quiescence during aging and start to differentiate (34). Precedence of such an interpretation comes from mouse models, where lymphopenia-induced accelerated homeostatic proliferation leads to the acquisition of memory phenotypes; moreover, virtual memory T cells in response to cytokines accumulate with age (9, 35, 36).

Epigenetic evidence of age-associated differentiation was more pronounced for CD8 T cells than CD4 T cells (16). A possible explanation could be that central memory or stem-like memory CD8 T cells revert back to a largely naïve phenotype and therefore contaminate the naïve compartment while central memory CD4 T cells do not (37). Of note, such an explanation would imply that the age-associated loss in naïve CD8 T cells is even larger than generally assumed. Indeed, CD8 naïve T cells appear to be more differentiated than naïve CD4 T cells in young adults. However, previous studies of TCR diversity have shown that the loss in diversity is essentially similar for naïve CD4 and CD8 T cells (10). More importantly, the observation of age-associated greater differentiation was not limited to naïve T cells, but was also observed for central memory CD8 T cells. Alternatively, different control mechanisms of homeostatic proliferation may contribute to the distinct behaviors of these two subsets. Naïve and central memory CD4 and CD8 T cells live in the same niches formed by fibroblast reticular cell networks, where they are exposed to IL7. Regulatory regions of IL7R have recently been described to lose accessibility with age (16). In our study, IL7R was equally less accessible in CD4 T cells from old adults. However, naïve CD8 T cells may receive more consistent activation signals from recognizing abundantly expressed MHC class I molecules than CD4 T cells that depend on the recognition of MHC class II molecules. Moreover, expression of receptors for homeostatic cytokines differ. In particular, CD8 T cells are responsive to IL-15 in addition to IL-7. In the mouse, IL-15 accounts for the differentiation into virtual memory T cells that are mostly of the CD8 phenotype (18, 38).

The finding of epigenetic differentiation signatures is consistent with previously described functional features of T cells from old adults. Naïve T cells from old adults have lost some of their developmental plasticity, in part due to increased BATF and IRF4 activity (39, 40). Moreover, age-associated microRNA patterns resemble differentiation signatures (41, 42). Whether differentiation during homeostatic proliferation accounts for the decline in naive T cell populations and here particularly naïve CD8 T cells is unclear. In our epigenetic analysis, we did not see patterns of cellular senescence that could impair proliferation. It is possible that partial activation renders cells more susceptible to apoptosis, resulting in an eventual loss of naïve CD8 T cells.

In addition to these differentiation-resembling epigenetic changes, T cells from old adults exhibited losses in chromatin accessibility that were distinct for CD4 and CD8 T cells. ZBTB family binding sites were partially closed in CD4 T cells with aging while NRF1 and YY1 binding sites were less accessible in CD8 T cells. These age-associated changes epigenetic changes occurred in T cell subsets irrespective of their differentiation state. Reduced accessibilities appeared to be due to decreased expression of the respective TF. Transcripts of the CD4 lineage determining TF THPOK (ZBTB7B) decline with age. The decline may account for the expression of CD8α in some CD4 T cells with age, in particular in TEMRA populations. Other functional implications of this decline are unknown, however, it appears to be unlikely that the decline confers a survival advantage for naïve CD4 T cells.

Transcripts for NRF1 and YY1 also decline with age, but much more so for CD8 than CD4 T cells. The decline in YY1 may contribute the age-associated loss in miR-181a because YY1 is one of the major regulators of this microRNA (43). NRF1 and, to a lesser extent, YY1 have been linked to the transcriptional control of genes involved in mitochondrial function and biogenesis (44, 45). The targets of NRF1 include genes encoding subunits of the five respiratory complexes (46). As previously reported, NRF1 regulates the expression of mitochondrial respiratory chain complex genes by targeting their promoter regions (19). NRF1 has also been reported to regulate genes involved in assembly of the mitochondrial ribosomes (47). In that study, NRF1 recruited SIRT7, as a component of the mitochondrial unfolded protein response, to the proximal promoters of mitochondrial ribosomal proteins (MRPs) and mitochondrial translation factors to attenuate translation and restore protein homeostasis. In our study, we observed reduced accessibility to ribosomal protein promoters including MRPs in CD8 but not in CD4 T cells from older individuals. Correspondingly, transcription of ribosomal proteins is decreased significantly in CD8 T cells with age. In particular, MRPs transcripts were highly decreased in naïve CD8 T cells from older adults; a lesser decrease of MRP transcripts was also seen in naïve CD4 T cells. Decrease in MRP expression therefore appears to be a general feature of T cell aging, but it is more pronounced in CD8 T cells.

Several observations have supported the general concept that a low rate of translation plays a protective role in aging (48). Reduced protein synthesis with aging has been described in variety of cell types and tissues and organisms (49, 50). Depletion of ribosomal proteins or translation factors can significantly extend lifespan in yeast, worms, and flies (48, 51). It is therefore unclear whether the reduced accessibility to NRF1 and YY1 and the associated decline in ribosomal proteins are primary defects, possibly due to the reduced expression of the TFs, or protective responses, e.g., the mitochondrial unfolded protein response attenuates translation to restore proteostasis (47). In the latter case, CD8 T cells would have increased mitochondrial dysfunction. Alternatively, reduced mitochondrial translation could impair mitochondrial biogenesis and function, possibly causing mitochondrial proteostasis, and be therefore detrimental to cell function.

Collectively, our studies have identified age-associated epigenetic signatures that differ in CD4 and CD8 T cells and that may explain their different behaviors in T cell homeostasis and T cell aging. The chromatin accessibility maps indicate that naïve CD8 T cells are more at risk of not maintaining quiescence, therefore entering differentiation and losing stemness more easily with age. Moreover, they indicate defects in basic cellular and mitochondrial maintenance pathways, again more in CD8 T cells. These aging features may be interrelated or independently contribute to the accelerated loss of naïve CD8 T cells with aging.

## Data Availability Statement

ATAC-seq data for CD4 T cell subsets have been deposited in SRA with BioProject accession code PRJNA478249. ATAC-seq data for CD8 T cell subsets and RNA-seq data for NRF1 WT and knockdown T cells are available in the Database of Genotypes and Phenotypes, with accession code phs001187.v1.p1, RNA-seq data of CD4 and CD8 T cells subsets used in this study are deposited in SRA with accession code PRJNA638216.

## Ethics Statement

The studies involving human participants were reviewed and approved by Stanford Institutional Review Board. The patients/participants provided their written informed consent to participate in this study.

## Author Contributions

BH, RJ, CW, and JG designed research and analyzed data. BH, SL, CG, ZY, and XL performed the experimental work. LT performed statistical analysis. BH, RJ, CG, CW, and JG wrote the manuscript. All authors contributed to the article and approved the submitted version.

## Funding

This work was supported by the National Institutes of Health (R01 AR042527, R01 HL117913, R01 AI108906, R01 HL142068, and P01 HL129941 to CW and R01 AI108891, R01 AG045779, U19 AI057266, and R01 AI129191 to JG), Merit Review Award I01 BX001669 from the United States (U.S.) Department of Veterans Affairs and with resources and the use of facilities at the Palo Alto Veterans Administration Healthcare System. The content is solely the responsibility of the authors and does not necessarily represent the official views of the National Institutes of Health.

## Conflict of Interest

The authors declare that the research was conducted in the absence of any commercial or financial relationships that could be construed as a potential conflict of interest.
