# Corrigendum: Distinct Age-Related Epigenetic Signatures in CD4 and CD8 T Cells

**DOI:** 10.3389/fimmu.2022.911132

**Published:** 2022-04-27

**Authors:** Bin Hu, Rohit R. Jadhav, Claire E. Gustafson, Sabine Le Saux, Zhongde Ye, Xuanying Li, Lu Tian, Cornelia M. Weyand, Jörg J. Goronzy

**Affiliations:** ^1^Division of Immunology and Rheumatology, Department of Medicine, Stanford University, Stanford, CA, United States; ^2^Department of Medicine, Palo Alto Veterans Administration Healthcare System, Palo Alto, CA, United States; ^3^Department of Biomedical Data Science, Stanford University, Stanford, CA, United States

**Keywords:** ribosomal proteins, chromatin accessibility, epigenetics, T-cell, aging, T-cell homeostasis

In the original article, there was an error. The wrong accession number was given for the data deposited in the NCBI SRA repository. A correction has been made to the **Data Availability Statement**:

“ATAC-seq data for CD4 T cell subsets have been deposited in SRA with BioProject accession code PRJNA478249. ATAC-seq data for CD8 T cell subsets and RNA-seq data for NRF1 WT and knockdown T cells are available in the Database of Genotypes and Phenotypes, with accession code phs001187.v1.p1, RNA-seq data of CD4 and CD8 T cells subsets used in this study are deposited in SRA with accession code PRJNA638216.”

The authors apologize for this error and state that this does not change the scientific conclusions of the article in any way. The original article has been updated.

## Publisher’s Note

All claims expressed in this article are solely those of the authors and do not necessarily represent those of their affiliated organizations, or those of the publisher, the editors and the reviewers. Any product that may be evaluated in this article, or claim that may be made by its manufacturer, is not guaranteed or endorsed by the publisher.

