# Supplementary material for: Distinct Age-Related Epigenetic Signatures in CD4 and CD8 T Cells
Source: Front Immunol. 2020 Nov 11;11:585168. doi: 10.3389/fimmu.2020.585168 (PMC7686576; doi:10.3389/fimmu.2020.585168)
Supplement: Supplementary file 1 [file DataSheet_1.docx]

**Distinct age-related epigenetic signatures in CD4 and CD8 T cells**

Bin Hu^1,2^, Rohit R. Jadhav^1,2^, Claire E. Gustafson^1,2^, Sabine Le Saux^1,2^, Zhongde Ye^1,2^, Xuanying Li^1,2^, Lu Tian^3^, Cornelia M. Weyand^1,2^, Jörg J. Goronzy^1,2*^

^1^ Division of Immunology and Rheumatology, Department of Medicine, Stanford University, Stanford, CA, United States.

^2^ Department of Medicine, Palo Alto Veterans Administration Healthcare System, Palo Alto, CA, United States.

^3^ Department of Biomedical Data Science, Stanford University, Stanford, CA, United States

**Supplemental Material**

This file includes: Figure S1 to S11

**Figure S1**

**
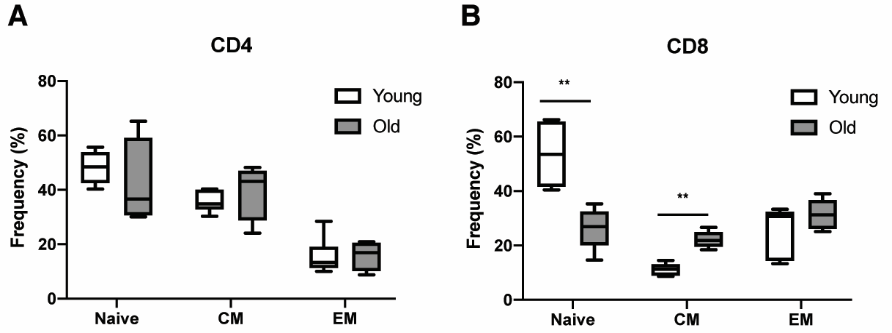
**

**Figure S1. Frequencies of CD4 and CD8 subsets in young and old individuals.** Frequencies of CD4 and CD8 naïve, central memory (CM) and effector memory (EM) T cells from young and older individuals are shown as box plots. Comparisons were done by two-tailed t-test; ** p < 0.01.

**Figure S2**

**
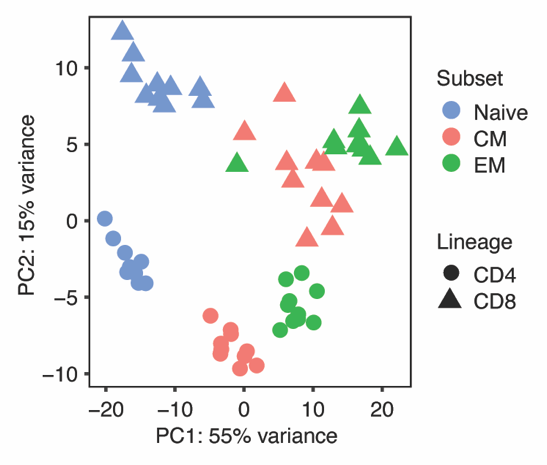
**

**Figure S2. Principal component analysis on ATAC-seq data**

Scatter plot shows the sample clustering based on PC1 and PC2 identified in Figure 1A and 1B, colors indicate the differentiation states and shapes show the lineages.

**Figure S3**

**
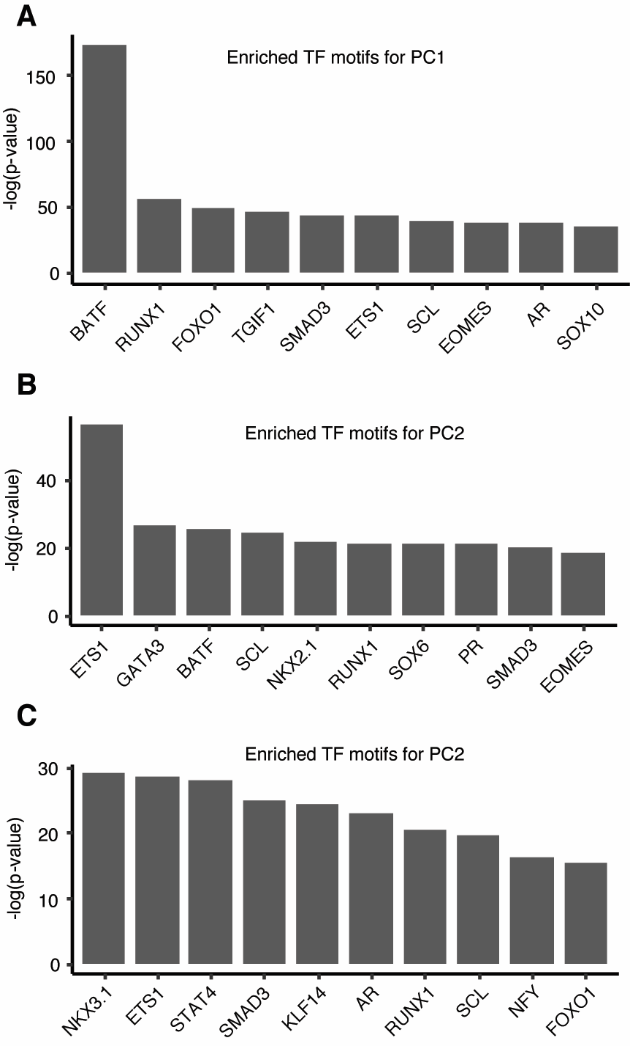
**

**Figure S3. Transcription factor networks of principal components.** Bar graphs show the top 10 transcription factor motifs enriched at sites loaded for each PC (Figure 1A - C) as determined by HOMER; y-axis shows the significance of TF binding motif enrichment.

**Figure S4**

**
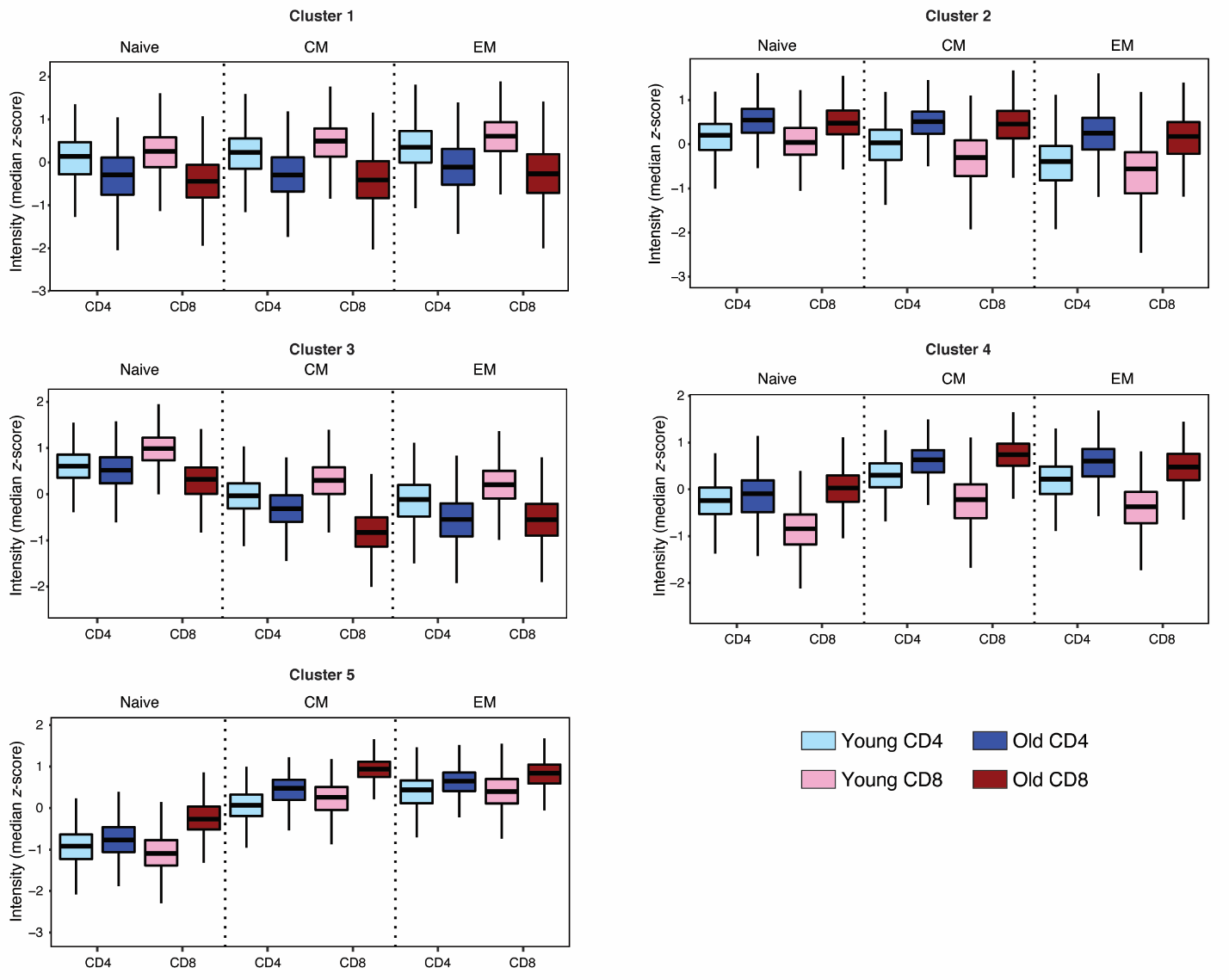
**

**Figure S4. Peak intensities of sites differentially accessible with age.** Box plots show the intensities of peaks for each cluster identified in Figure 3C. The intensity was calculated based on the *z*-score of the median accessibility for each group.

**Figure S5**

**
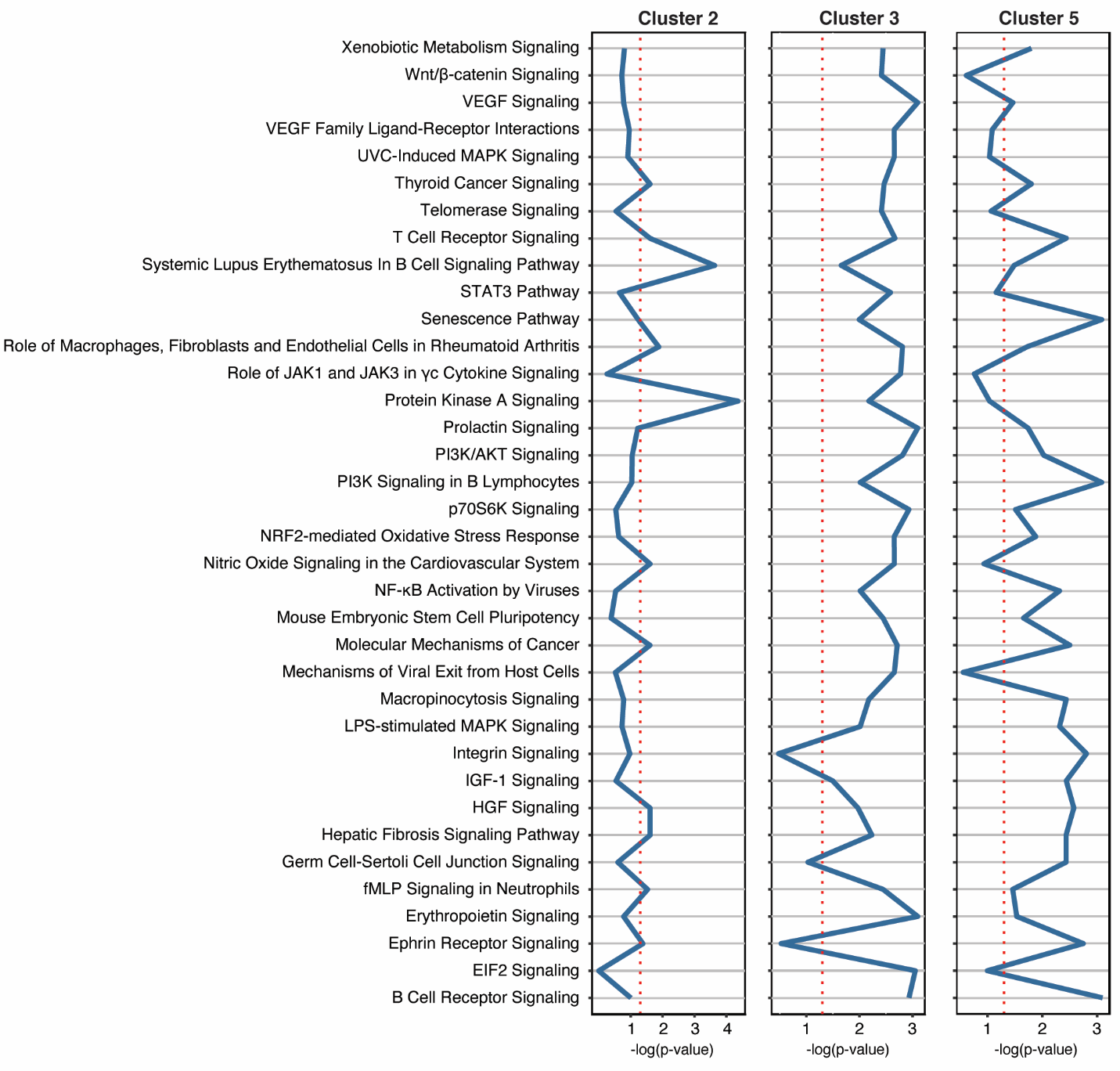
**

**Figure S5. Pathway analysis of genes regulated by differentially accessible sites.** Sites included in each cluster of the heat map shown in Figure 3C were assigned to genes by GREAT and the most enriched canonical pathways by Ingenuity Pathway Analysis for each gene sets were identified. X-axis shows the adjusted p-value, red dash line indicates adjusted p-value of 0.05. Genes in Clusters 1 and 4 did not have a significant enrichment for a pathway.

**Figure S6**


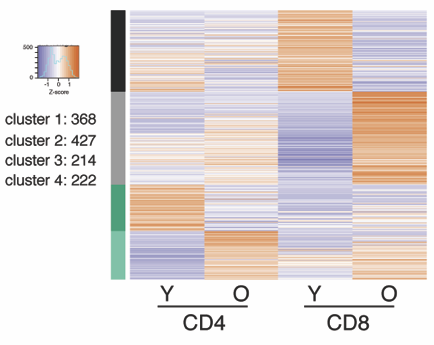


**Figure S6. *k*-means analysis of genes differentially expressed in aging.** *k*-means analysis of genes differentially expressed in naïve CD4 and CD8 aging as identified in Figures 4A and 4B. Results are shown as heat plot with each horizontal line representing a gene differentially expressed with age. Columns represent CD4 and CD8 naïve T cell subsets from young and old adults; colors *z*-scores of transcript level.

**Figure S7**


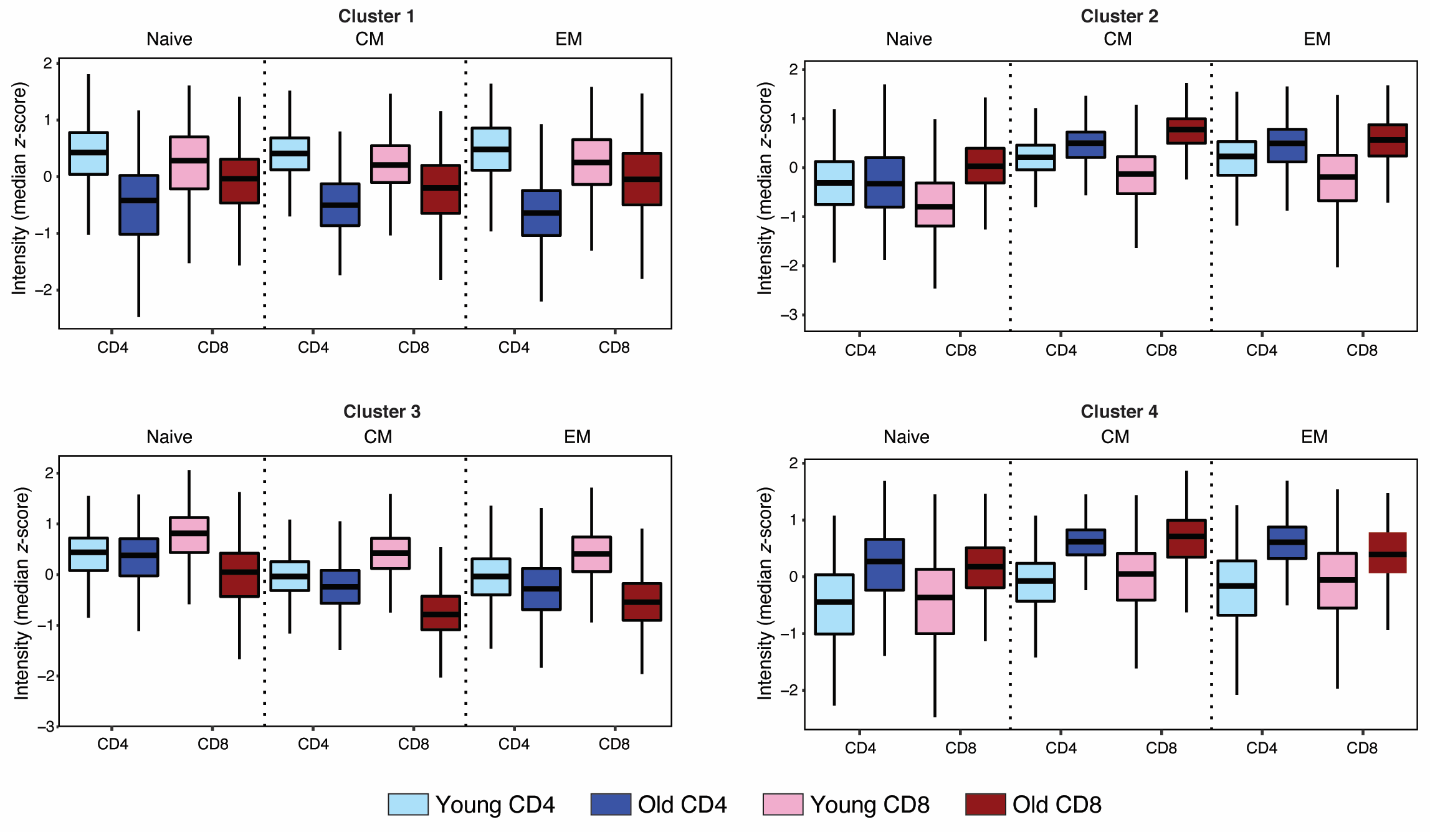


**Figure S7. Chromatin accessibilities of peaks preferentially changing with age in CD4 or CD8 T cells.** Box plots show the intensities of peaks within each cluster identified in Figure 5A. Intensities were calculated based on the *z*-score of the median accessibility for each group.

**Figure S8**


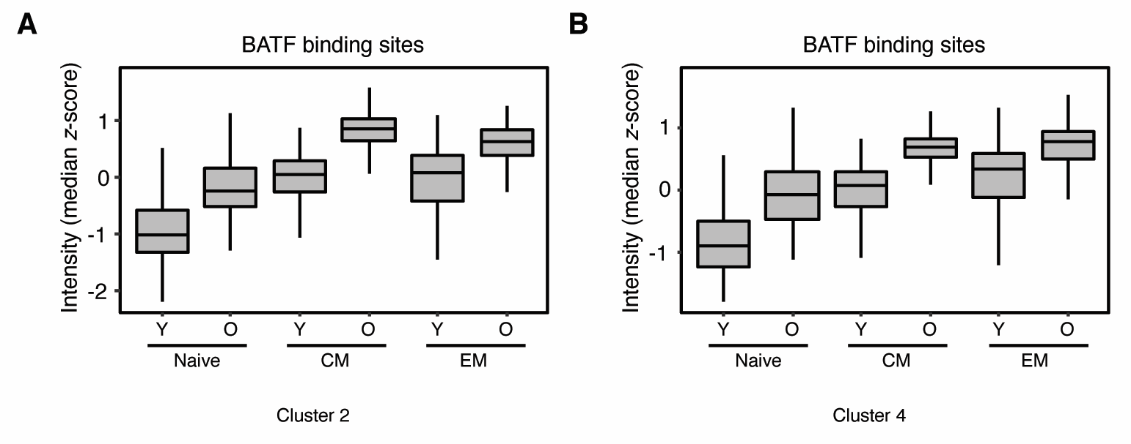


**Figure S8. Peak intensities at BATF motifs of sites with age-associated changes.** **(A and B)** Box plots show the peak intensities of BATF motifs in peaks within Cluster 2 (A) and Cluster 4 (B) shown in Figure 5A. The motif intensity was calculated based on the *z*-score of the median accessibility for each group.

**Figure S9**

**
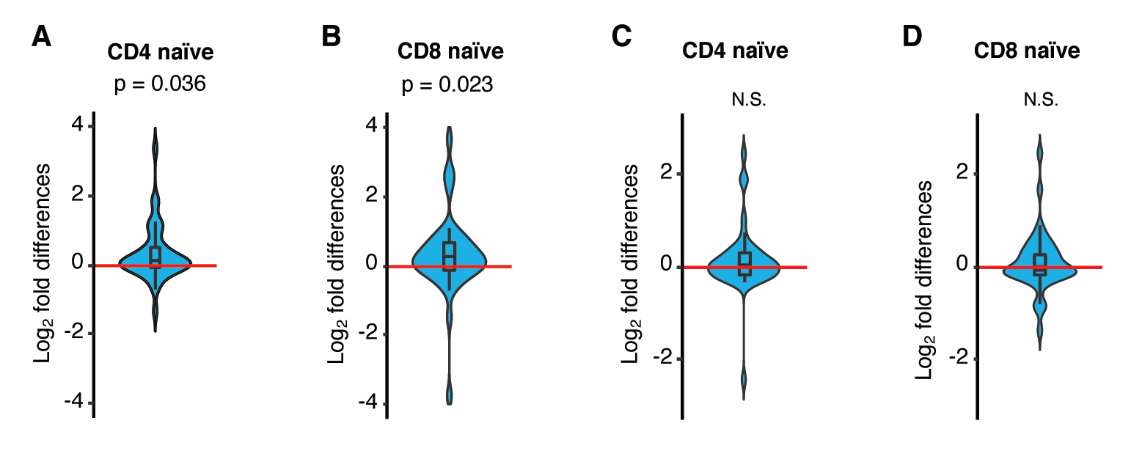
**

**Figure S9. Transcript expression of TF family members in naïve T cells from young and old adults. (A** and **B)** Violin plots of the fold differences of bZIP family member transcripts comparing naïve CD4 (A) and CD8 (B) T cells from young and old adults. Gene list for bZIP family member obtained from reference 27. **(C** and **D)** Violin plots of the fold differences of ZBTB family member transcripts comparing naïve CD4 (E) and CD8 (F) T cells from young and old adults. Gene list for ZBTB family proteins was obtained from reference 26. Positive value indicates higher expression with age. Statistical analysis by Wilcoxon rank sum tests.

**Figure S10**

**Figure S10. Enrichment for biologic processes in genes with age-related differentially accessible sites.** Genes within 10 kb of a differentially accessible region were identified using HOMER. Enrichment analysis of genes within clusters 1, 2 and 4 as defined in Figure 5A was performed using the DAVID Functional Annotation Tool. The five most significantly enriched biological processes are shown as determined by a modified Fisher Exact test (left). ATAC-seq signal tracks for genes within indicated clusters representative for biological processes (right).

**Figure S11**

**
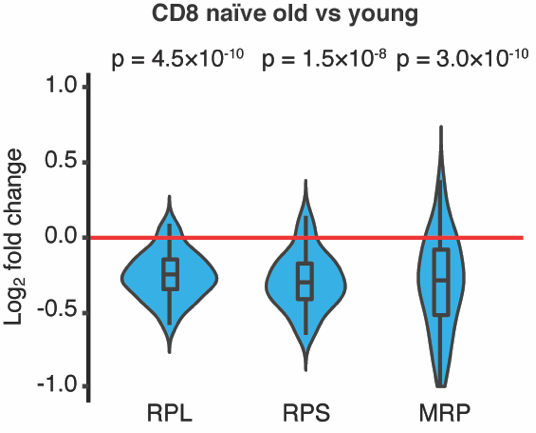
**

**Figure S11. Transcript levels of ribosome proteins decline in naïve CD8 T cells with age.**

Violin plots show the fold differences of ribosome protein (large subunit, RPLs; small subunit, RPSs; and mitochondrial, MRPs) transcripts comparing CD8 T cells from young and old adults. Transcript data are from a previously reported RNA-seq data set of naïve CD8 from three young and three old adults (dbGaP accession #: phs001187.v1.p1). Statistical analysis by Wilcoxon rank sum tests.
